# NRF2 as an Emerging Therapeutic Target

**DOI:** 10.1155/2017/8165458

**Published:** 2017-01-31

**Authors:** Ian M. Copple, Albena T. Dinkova-Kostova, Thomas W. Kensler, Karen T. Liby, W. Christian Wigley

**Affiliations:** ^1^MRC Centre for Drug Safety Science, Department of Molecular and Clinical Pharmacology, University of Liverpool, Liverpool L69 3GE, UK; ^2^Division of Cancer Research, School of Medicine, University of Dundee, Dundee DD1 9SY, UK; ^3^Department of Pharmacology and Chemical Biology, University of Pittsburgh, Pittsburgh, PA 15261, USA; ^4^Department of Pharmacology and Toxicology, Michigan State University, East Lansing, MI 48824, USA; ^5^Reata Pharmaceuticals Inc., 2801 Gateway Drive, Irving, TX 75063, USA

The transcription factor nuclear factor erythroid 2 related factor 2 (NRF2) is the master regulator of the basal and inducible expression of a large network of cytoprotective genes. As a result, NRF2 plays a key role in antagonising a range of pathological insults including reactive oxygen species and toxic xenobiotics [1]. Consistent with this, dysregulation of NRF2 signalling is associated with an increased susceptibility to and/or accelerated progression of a range of experimental diseases in mice. In recent years, NRF2 has shown promise as a novel therapeutic target in human diseases, particularly those with underlying oxidative and inflammatory stress components [2]. Indeed, several NRF2 inducers have recently entered the clinic and a number of pharmaceutical companies have NRF2-based programs. The goal of this special issue is to highlight the promise of NRF2 as a novel therapeutic target in a number of disease settings and in turn foster further research in this burgeoning field.

NRF2 is dysregulated in numerous human pathologies and thus represents an attractive drug target. A number of electrophilic NRF2 activators, including naturally occurring isothiocyanates and semisynthetic triterpenoids, are currently in clinical trials, and an oral preparation of dimethyl fumarate (DMF, Tecfidera) is used in clinical practice to reduce disease progression in patients with relapsing remitting multiple sclerosis. In multiple sclerosis, neuronal degeneration is linked to glutamate-induced excitotoxicity and oxidative stress, and it has been reported that DMF protects cells against the neurotoxicity of glutamate [3, 4]. In this special issue, C. Hoffmann et al. show that one of the consequences of exposure of cells to DMF is an increase in glutathione recycling by induction of the NRF2 transcriptional target glutathione reductase, the enzyme which catalyzes the regeneration of oxidized glutathione (GSSG) to its reduced (GSH) form. Curiously, however, inhibition (genetic or pharmacological) of glutathione reductase has a synergistic protective effect to that of DMF. The authors further show that this protection correlates with activation of a number of NRF2-dependent genes. These findings confirm the critical cytoprotective role of glutathione, the most abundant intracellular antioxidant, and illustrate the versatility and robustness of NRF2-mediated cytoprotection.

Two papers in this special issue highlight the protective effect of NRF2 in the eye. K. Takayama et al. describe a role for NRF2 in protection against blue light retinal pigment epithelial (RPE) cell damage. Specifically, the authors show that blue light exposure (450 nm) stimulates reactive oxygen species generation and NRF2 signalling in a human RPE cell line, and that primary RPE cells from transgenic Nrf2 knockout mice are more sensitive to blue light induced cell death compared with cells from wild type mice.

Using a pharmacological approach, X. Xu et al. show that the flavonoid apigenin protects a human RPE cell line against tert-butyl hydroperoxide-induced oxidative stress and cell death via the stimulation of NRF2 signalling, with the protective effect abolished following siRNA knockdown of NRF2. The findings of these studies are consistent with previous work showing that aged Nrf2 knockout mice develop ocular pathology with similar features to human age-related muscular degeneration, a leading cause of blindness [5]. These and other preclinical studies highlight the potential of NRF2 as a novel therapeutic target in ocular disease, and may inform future clinical trials in this area.

Angiotensin II (Ang II) is a vasoconstrictive hormone and a key component of the renin-angiotensin system, which regulates vascular tone and blood pressure. Although largely associated with effects on vascular smooth muscle and kidney epithelia, receptors for Ang II are present in testes, suggesting a potential role in male fertility. S.-J. Wang et al. have contributed with an interesting study assessing the effects of Ang II on testicular cell viability and the potential impact of Nrf2 induction with the isothiocyanate sulforaphane. In wild type and Nrf2 knockout mice, treatment with Ang II was associated with testicular weight loss, oxidative and endoplasmic reticulum stress, inflammation, and apoptotic cell death. Treatment with sulforaphane was protective in wild type mice but not in those lacking Nrf2, demonstrating the Nrf2-dependence of the efficacy. These results further illustrate the importance of Nrf2 in male fertility and provide additional insights into the influence of Ang II signalling on testicular health and function.

Our expanding knowledge of the regulatory influences on NRF2 activity continues to inform new strategies for targeting the transcription factor beyond the use of electrophilic agents that have the ability to modify critical cysteine residues in KEAP1, the redox-sensitive repressor of NRF2. Indeed, compounds and peptides that disrupt the binding interface between NRF2 and KEAP1 have been described [6] and may represent an important alternative therapeutic approach given the propensity of some electrophiles to react with unintended targets. In their review, H. Yukitake et al. highlight the role of macrophage migration inhibitory factor (MIF) as a regulator of NRF2-driven gene expression. The authors describe how a chemical screening process identified the cardioprotective agent BTZO-1, which was subsequently found to augment the expression of NRF2-regulated genes in a MIF-dependent manner, similarly to recombinant MIF protein. In proposing that MIF is an alternative sensor for electrophilic NRF2 activators, H. Yukitake et al. highlight the importance of appreciating the selectivity of small molecules that target NRF2 and other signalling pathways, particularly for understanding the role of NRF2 per se in the therapeutic effects of a given compound.

Zebrafish (*Danio rerio*) are an important and widely used vertebrate model organism for studies on development and gene function. Using this model, the Kobayashi laboratory has described previously the evolutionary conservation of Keap1-Nrf2 signalling in defence against oxidative and electrophilic stresses. Herein, V. T. Nguyen et al. describe the conduct of a microarray analysis of zebrafish embryos that either overexpressed Nrf2 or were challenged with small molecule activators of the pathway. These genetic and pharmacologic approaches demonstrate that additional functions of the Nrf2 pathway, namely, the regulation of protein turnover and glucose metabolism that have been observed in higher vertebrates, are conserved in zebrafish. These results provide further support for the notion that the key actions of Nrf2 extend far beyond, evolutionarily and functionally, its canonical actions of affecting xenobiotic metabolism. Indeed, actions on protein turnover and glucose metabolism may be central to the evolutionary development of this important signalling pathway.

In summary, this special issue highlights recent advances in our understanding of the regulatory mechanisms, physiological roles, and cytoprotective effects of NRF2 in a range of preclinical models. At present, the benefits and risks of modulating NRF2 pathway activity in patients are not fully understood, and it is known that NRF2 and KEAP1 may cross-talk with other signalling pathways, such as NF-*κ*B. Indeed, many NRF2 inducers directly influence the activities of these pathways, and thus it will be important to establish the true therapeutic value of modulating NRF2 per se in man. However, with an expanding number of compounds entering clinical trials, the field should be well-placed to answer these and other pertinent questions.
